# Specificity ratings for Italian data

**DOI:** 10.3758/s13428-022-01974-6

**Published:** 2022-09-26

**Authors:** Marianna Marcella Bolognesi, Tommaso Caselli

**Affiliations:** 1 Faculty of Modern Languages, Literatures and Cultures, University of Bologna, Bologna, Italy; 2 Faculty of Arts, CLCG, University of Groeningen, Groningen, The Netherlands

**Keywords:** Abstraction, Categorization, Human ratings, Specificity, Concreteness, Italian data

## Abstract

Abstraction enables us to categorize experience, learn new information, and form judgments. Language arguably plays a crucial role in abstraction, providing us with words that vary in specificity (e.g., highly generic: *tool* vs. highly specific: *muffler*). Yet, human-generated ratings of word specificity are virtually absent. We hereby present a dataset of specificity ratings collected from Italian native speakers on a set of around 1K Italian words, using the Best-Worst Scaling method. Through a series of correlation studies, we show that human-generated specificity ratings have low correlation coefficients with specificity metrics extracted automatically from WordNet, suggesting that WordNet does not reflect the hierarchical relations of category inclusion present in the speakers’ minds. Moreover, our ratings show low correlations with concreteness ratings, suggesting that the variables Specificity and Concreteness capture two separate aspects involved in abstraction and that specificity may need to be controlled for when investigating conceptual concreteness. Finally, through a series of regression studies we show that specificity explains a unique amount of variance in decision latencies (lexical decision task), suggesting that this variable has theoretical value. The results are discussed in relation to the concept and investigation of abstraction.

## Introduction

Any theory of meaning must accommodate different types of semantics. Yet, most research is focused on words denoting concrete, basic level concepts, such as *table*, *dog*, *banana* (McRae & Jones, 2013). Cognitive studies that take into account aspects associated with abstraction, typically focus on the comparison between concrete *vs*. abstract concepts, leaving aside their variation in specificity. These studies tend to show that words denoting concrete concepts are more easily recognized, recalled, learned, comprehended, and produced compared to words denoting abstract concepts (Hoffman, 2016 for a review). Taken together, these empirical findings are commonly referred to as “the concreteness effect” (e.g., Jessen et al., 2000).

However, the scientific literature reports also reversed effects where a processing advantage of abstract over concrete words is observed (Barber et al., 2013; Kousta et al., 2011; Vigliocco et al., 2014). This has been attributed to methodological issues that characterize some experimental studies focused on concreteness, and in particular to the selection of concrete and abstract stimuli. For example, “whereas most studies controlled for differences in frequency between concrete and abstract words, many of the studies above did not control for differences in familiarity, leading to comparisons between more familiar (and therefore easier to process) concrete (e.g., *artichoke*) and less familiar, abstract (e.g., *heresy*) words” (Vigliocco et al., 2014:1767).

Crucially, the words selected to investigate the concreteness effect may differ in specificity, namely in how inclusive the category of reference is. A category that is low in specificity is characterized only by a few features and is highly inclusive. A category that is high in specificity is characterized by several features and is therefore less inclusive. In this sense, a highly specific word, characterized by several features, is semantically richer than a non-specific word, because its semantic structure has a higher resolution. From a linguistic perspective, specificity can be operationalized in terms of word positioning in a lexical taxonomy: the more hypernyms a word has, the more it denotes a category that is high in specificity, and thus, it is placed toward the leaves of the taxonomy. For instance, the category *coffee table* can count many hypernyms (*table*, *furniture*, *object*, among others). On the other hand, the more hyponyms a word has, the more it denotes a category that is low in specificity, and thus, it is high in the taxonomy, toward the root nodes. For instance, the category *object* is low in specificity and has many levels of hyponyms.

Categorical specificity is a core construct in cognitive science (e.g., Rosch & Mervis, 1975; for a review: Cohen & Lefebvre, 2005). Traditionally, however, the mechanisms of semantic categorization and the characteristics of the hierarchical architecture of our semantic memory have been investigated by taking into account concrete concepts only (e.g., McRae & Jones, 2013). Yet, specificity characterizes both concrete and abstract concepts alike: *footwear* is a concrete conceptual category that includes the more specific category *sandal*, as much as *religion* is an abstract conceptual category that includes the more specific category *Buddhism*.

The influence of word specificity on word processing and its relationship with word concreteness remain to be properly understood. To address these goals, first specificity must be operationalized into a numeric variable so that it can be compared to other linguistic and psychological variables involved in abstraction, such as Concreteness, Familiarity, and Frequency. To tackle this objective, Bolognesi et al. (2020) operationalized specificity in numeric scores automatically extracted from a lexical resource (namely: WordNet; Miller, 1995) and correlated it to human-generated concreteness ratings (Brysbaert et al., 2014). Results showed that, although positively correlated, the two variables Concreteness and Specificity capture different phenomena: words can be concrete and specific (e.g., *Aspirin*, *muffler*, *pulpit*), concrete and generic (*substance*, *tool*, *construction*), abstract and specific (*ratification*, *Buddhism*, *forensics*) or abstract and generic (*law*, *religion*, *beauty*).

As a potential limitation of their study, Bolognesi and colleagues explain that WordNet is an encyclopedic dictionary, with wide lexicographic coverage of terms, which are linked to one another by means of various types of semantic relations that have been extracted from corpora and other lexical resources, or manually coded by lexicographers. Despite the cognitive principles that underpin and motivate the WordNet taxonomy and the semantic relations encoded therein, the specificity scores that can be extracted from this lexical resource are not directly generated by humans and they do not necessarily reflect the hierarchical relations between word meanings in the speakers’ minds. Thus, while the overall cognitive plausibility of the relations encoded in WordNet is supported by the scientific literature (Miller, 1998; Fellbaum, 1998), the actual entries and the hierarchical relations between them have not been directly produced by humans and lack cognitive validity. For instance, WordNet contains 39 hyponyms for the entry *insect*^1^, many of which may be unknown to most speakers. Therefore, it remains to be investigated to what extent the specificity scores extracted from WordNet actually reflect word specificity in the speakers’ mind.

To address this potential limitation, we hereby collect and present a dataset of human-elicited specificity ratings for more than 1000 Italian words (nouns, verbs, and adjectives). The human-generated specificity ratings are hereby correlated with specificity ratings automatically extracted from WordNet (Study 1). Then, we correlate our specificity ratings with human-generated concreteness ratings (Study 2). We further correlate the human-generated specificity ratings with two variables that previous studies have shown being related with Concreteness (e.g., Gilhooly & Loogie, 1980; Kousta et al., 2011). These are: 1. Age of acquisition, and 2. Affective content, operationalized into three dimensions (Valence, Arousal and Dominance). Moreover, to provide a comprehensive picture on the specificity hereby collected and their relation to abstraction, we run an additional set of correlation analyses between Specificity and three variables that are commonly controlled for, when selecting concrete and abstract stimuli, namely 1. Familiarity; 2. Frequency; and 3. Imageability (Study 3). While in Study 1 the comparison between specificity ratings extracted from WordNet and human-generated ratings is conducted on a dataset of nouns because WordNet encodes the hierarchical relation of category inclusion for nouns, in Study 2 and 3 the correlations are run on a more encompassing dataset of words that include nouns, verbs and adjectives. Finally, in Study 4 we provide a series of individual, simultaneous and incremental regressions in which we show how specificity (and the other psychological variables) predict chronometric data and in particular reaction times in a lexical decision task. This last study provides empirical support for the relevance of word specificity in lexical access.

Our research questions can be summarized as follows:

- Research Question 1: Does the categorical specificity that speakers have in mind correlate with specificity scores extracted from WordNet?
- Research Question 2: What is the relation between Concreteness and Specificity, when the two variables are compared through ratings elicited directly from speakers?
- Research Question 3: What is the relation between specificity and other lexical and psychological variables associated with or controlled while investigating Concreteness, namely Age of Acquisition, Affective content, Frequency, Familiarity and Imageability?
- Research Question 4: What is the role of Specificity in lexical access and therefore how do human-generated specificity scores predict chronometric data in a lexical decision task?

All data and materials are available at the following OSF repository: https://osf.io/ahf82/?view_only=c814286465dd4b119ce83bf8eb4c82fb.

## Theoretical background

“Abstraction” is originally a Latin word and can be literally translated as *pulled off from*. Abstraction, a hallmark of human cognition, is the ability to *pull off meaning from experience*. Abstraction processes are at the foundation of the ability to learn new information (e.g., Glenberg et al., 2011; Hinds et al., 2001; Mandler & McDonough, 1998; McGinnis & Zelinski, 2000), form judgments (e.g., Henderson et al., 2006; Klein et al., 1992; Ledgerwood et al., 2010; Wakslak, 2012), regulate behavior (e.g., Freitas et al., 2004; Fujita et al., 2006; Schmeichel et al., 2011), create and appreciate art (Witkin, 1983; Hackett, 2016).

Scholars from different fields refer to abstraction making different assumptions and sometimes embracing different definitions. While cognitive scientists usually refer to abstraction as the ability to think and talk in terms of conceptual categories that are detached from *perceptual* experiences, computer scientists tend to use abstraction as the ability to think and talk in terms of conceptual categories that have different degrees of inclusiveness, and are therefore detached from *specific* instances or examples. These two definitions capture different aspects of abstraction. We name these two variables, respectively, Concreteness and Specificity. Concreteness indicates the extent to which a concept (and the relative word) is associated with perceptual experience, whereas specificity indicates the extent to which a category is precise and detailed.

The following passages - (1) from scholars in cognitive science, (2) from a scholar in computer science/AI - show that these two aspects involved in abstraction can be intuitively conflated with one another.

1. *Lower levels of abstraction (i.e., higher levels of concreteness) capture thoughts that are more specific, detailed, vivid, and imageable *(e.g., Strack, Schwarz, & Gschneidinger, 1985)*, often encompassing readily observable characteristics (e.g., furry dog, ceramic cup; Medin & Ortony,* 1989*). Higher levels of abstraction (i.e., lower levels of concreteness), on the other hand, include fewer readily observable characteristics and therefore capture thoughts that are less imageable (e.g., friendly dog, beautiful cup).* (Burgoon et al., 2013: 503).
2. [Starting from the concrete notion of bridge], *humans can easily understand extended and metaphorical notions such as “water bridges,” “ant bridges,” “the bridge of a song,” “bridging the gender gap,” “a bridge loan,” “burning one’s bridges,” “water under the bridge,” and so on. […] One makes an abstraction of a concept when one extends that concept to more general instances, ones that are more removed from specific entities, as in the examples of “bridge”.* (Mitchell, 2021).

In (1) the authors assume that conceptual categories that are highly specific and detailed are also highly concrete and imageable. Yet, *entropy*, for instance, is a quite specific and precise concept, but is not vivid, imageable or concrete. In (2) the author implies that something like “a bridge over gender gap” (which is an abstract concept) is more general, and thus more detached from specific entities, compared to “a bridge over a river” (which is a concrete concept). Yet, a bridge over gender gap is a very specific (but abstract!) concept, applicable to a restricted type of situations. These examples show how Concreteness and Specificity can be merged with one another, under the (undemonstrated) assumption that abstract concepts are also more generic than concrete concepts, and that concrete concepts are also more specific than abstract ones.

Decoupling Concreteness and Specificity is a crucial step, needed for understanding the mechanisms of abstraction and semantic categorization, and explaining how “we could ever achieve the higher-order generalizations on which so much of our semantic processing relies.” (Patterson et al., 2007:977).

In order to understand the relation between Specificity and Concreteness, the two factors need to be operationalized into comparable variables. Concreteness is typically operationalized through concreteness ratings, elicited directly from speakers. Datasets including concreteness norms are now available for several languages. To name just a few recent ones, Montefinese et al. (2014) for Italian, Guasch et al. (2016) for Spanish, Soares et al. (2018) for Portuguese, Yao et al. (2017) for Chinese, Bonin et al. (2018) for French, Peti-Stantić et al. (2021) for Croatian. For English, Brysbaert et al. (2014) is among the most widely used. In this dataset, for instance, on a scale from 1 to 5 speakers judge *carrot* to have an average concreteness score of 5.0 (maximally concrete) while *hope* has a concreteness score close to 1.19 (extremely abstract). Concreteness ratings are extensively used in various disciplines, such as cognitive science (Ferreira et al., 2015; Siakaluk et al., 2008), experimental psychology (Kaushanskaya & Rechtzigel, 2012; Pexman et al., 2017), psycholinguistics (Ferreira et al., 2015; Van Rensbergen et al., 2015), and cognitive linguistics (Dunn, 2015).

Specificity, on the other hand, has never been operationalized into human-generated data, to the best of our knowledge. In the present study, we collect and analyze a dataset of human-generated ratings of word specificity in Italian, based on the words listed in the ANEW dataset (Montefinese et al., 2014, available here: https://osf.io/eu7a3/), containing human judgments about Concreteness, Affect, Familiarity, Frequency, Imageability, and the ITAoA dataset (Montefinese et al., 2019, available here: https://osf.io/3trg2/), containing human judgements about a word’s average Age of Acquisition.

In relation to the research questions, our hypotheses can be summarized as follows:

- Hp1: We expect to find a high, positive correlation between the Italian specificity scores extracted from Open Multilingual WordNet (OMW; Bond & Paik 2012) on the basis of the procedures and formulas originally used by Bolognesi et al. 2020, and the Italian specificity ratings elicited from human participants. This will be expected, given the cognitive underpinnings of the semantic relations encoded in the WordNet taxonomy.
- Hp2: Overall, we expect to find a positive correlation between human-generated concreteness and specificity scores in Italian, as we previously found on English data, using specificity scores extracted from WordNet (Bolognesi et al., 2020). We also expect this correlation to be not particularly high. This would be in line with the idea that Concreteness and Specificity capture different aspects of abstraction, which are only partially correlated with one another.
- Hp3: In relation to the age of acquisition ratings, we expect to find that words with medium levels of specificity scores are associated with lower ages of acquisition ratings, while words with high and low specificity scores are associated with higher age of acquisition ratings. This would be in line with Rosch and Mervis theories of basic level categorization (Rosch et al., 1976), in which the authors claim that words associated with medium levels of specificity (basic level categories) are also learned earlier (Mervis & Crisafi, 1982; Blewitt, 1983; Greco & Daehler, 1985). Thus, when looking at the distribution of age of acquisition ratings as a function of specificity ratings, we expect to find a U-shaped plot, in which very specific and very generic words correspond to higher age of acquisition scores, while words with medium scores of specificity would be associated with low age of acquisition scores.
  Judgments about the affective content of word meanings are collected through three dimensions: Valence (the polarity of emotional activation), Arousal (the intensity or degree of excitement caused by an emotion), and Dominance (the degree of control an individual feels while experiencing an emotive state). These three dimensions are related in different ways to one another (see Montefinese et al., 2014) and the relation of each of these three dimensions to Concreteness is debated. Montefinese and colleagues, for instance, found no correlation between each of the three dimensions of affective content and word concreteness. Vigliocco et al. (2014) provided evidence that abstract words tend to have more affective associations (either positive or negative) than concrete words. This would be the case even after imageability and context availability are controlled. In other words, more valenced (both, either positive or negative) and arousing words tend to be more abstract, whereas neutral words tend to be more concrete. In relation to word specificity, it is not easy to formulate hypotheses for each of the three dimensions involved in affection. In line with previous findings on the relation between Concreteness and affective content, by Montefinese et al. (2014), we expect to find no correlation between Specificity and the three dimensions of affective content.
  In relation to Frequency and Familiarity, we assume that words with medium levels of specificity correspond to basic level categories rather than superordinate or subordinate categories. Based on previous literature (see Hajibayova, 2013 for a review), these words are usually shorter, morphologically simpler, and learned earlier by children (e.g., *cat*, *ball*, *run*). These are also the peculiarities of highly frequent words. We therefore hypothesize that words associated with medium levels of specificity are more likely to be perceived as more familiar, and to be used more frequently, compared to highly specific and highly generic words.
  In relation to Imageability, we hypothesize that we may find a pattern that reflects the correlation between Specificity and Concreteness, based on the argument that Imageability and Concreteness are highly correlated with one another.
- Hp4: We expect specificity to play a role in lexical access and therefore to explain a substantial amount of variance in chronometric data such as reaction times collected during a lexical decision task. We base our hypothesis on empirical evidence showing that semantically rich words are processed faster than semantically poor words (Pexman et al., 2002, 2003; Grondin et al., 2009). Which words we assume to be semantically rich is hereby explained. According to classic cognitive studies in semantic categorization (e.g., Rosch et al., 1976) the basic level of semantic categorization includes categories that are maximally distinct from one another. It is at this level that categories carry more information, in the sense that the properties of the category members at this level are maximally informative (maximized cue validity, Rosch et al., 1976:384–385). We therefore expect basic level categories, i.e., words with medium levels of specificity, to be processed faster than highly specific or highly generic words. This implies a quadratic relation (U-shaped) between specificity and chronometric data.

The rest of the paper is organized as follows. In Study 1, we address the first research question, describing the methods used for the collection of the human specificity ratings and the results obtained by correlating the data with specificity scores extracted from WordNet. In Study 2, we address the second research question, describing methods and results obtained by comparing human-generated concreteness and specificity scores. In Study 3, we illustrate methods and results for the investigation of the relation between specificity scores and age of acquisition ratings, affective ratings, Familiarity, Frequency and Imageability. In Study 4, we address the role of specificity in lexical processing. Finally, we provide a general discussion of our findings.

## Study 1: Does the categorical specificity that speakers have in mind correlate with specificity scores extracted from WordNet?

### Methods

#### Collecting human-generated specificity ratings

Specificity ratings were collected online, through Google Forms platform, for all the 1186 target words (verbs, adjectives, and nouns) in Italian included in the dataset collected by Montefinese et al. (2014). This dataset is based on a translation into Italian of the words included in the original English dataset of affective norms ANEW (Bradley & Lang, 1999).

To elicit specificity rating, we adopted the Best-Worst Scaling method (henceforth BWS, Louviere et al., 2015). The BWS method has been shown to be particularly effective for the collection of norms when compared to other response formats, such as rating scaling (Hollis & Westbury, 2018).^2^ In this experimental design, participants were presented with a list of *m* tuples of four words each, all belonging to the same part of speech (e.g., either four nouns, or four verbs, or four adjectives) in *N,* i.e.*,* the total number of words for which the ratings must be collected in a trial. For each trial, namely, within each tuple, participants had to select the most specific and the least specific word.

To generate the *m* 4-tuples per part of speech, we used a publicly available implementation^3^ for BWS. The scripts generate distinct 4-tuples for each part of speech in a random way guaranteeing that each word is seen in eight different 4-tuples and that no word appears more than once in each tuple. In particular, for the nouns (*N* = 843) we generated 1686 distinct 4-tuples, for the verbs (*N* = 58) 116 4-tuples, and, finally, for the adjectives (*N* = 285) 570 unique 4-tuples. Each word appears in multiple tuples and is therefore evaluated in relation to multiple other words.

The tuples were then grouped into lists. The number of tuples presented to each participant (thus in each list) ranged from 30 to 40, depending on the part of speech and the words in the dataset. We prepared 15 lists containing tuples of adjectives, 43 lists containing tuples of nouns, and four lists containing tuples of verbs. All 60 lists were distributed to participants.

The 352 (male and female) participants who took part in the task were Italian native speakers, enrolled at various BA programs in the Humanities, recruited by two research assistants (BA and MA internship students) through the Experimental Lab, University of Bologna, Italy. Participants were contacted through university students’ groups on Facebook, and took part in the data collection task on a voluntary basis. Each participant could fill up to three lists and their voluntary participation in the research was not rewarded with a physical gift or course credit, but with our commitment to follow up and update them with the results of the study. The data collection was performed in accordance with the ethical standards as laid down in the 1964 Declaration of Helsinki and its later amendments, and in compliance with the requirements of the Ethics Committee of the hosting institution (University of Bologna, Italy).

Each tuple has been annotated by ten participants following the findings of Kiritchenko and Mohammad (2017), and Mohammad (2018). Once the data were collected, the specificity ratings were generated by applying the *counting procedure* (Orme 2009). An alternative procedure (value scoring, Hollis 2018) was run for comparison, and the results obtained with each of the two procedures showed a very high correlation (> .90). In the counting procedure, a word specificity rating corresponds to the percentage of times the term was chosen as most positive minus the percentage of times the term was chosen as most negative. With this procedure it is then possible to rank the words according to specificity values on a scale between – 1 (least specific) and 1 (most specific), as indicated in Table 1.

**Table 1:** Trial for collecting adjective specificity scores through BWS

	LA PIU’ SPECIFICA (most specific)	LA PIU’ GENERICA (most generic)
Riluttante (reluctant)	x	
Sorpreso (surprised)		
Timido (shy)		
Emotivo (emotional)		x

Instructions were provided in electronic format at the beginning of the task, in Italian, together with an informed consent form (see OSF repository). After a brief introduction and indication of age range, gender and geographical origin, participants were shown a hypothetical trial with a hypothetical response. For instance, a list containing adjectives would display a trial like the example provided in Table 1.

Under the explanatory trial a definition of “specificity” adapted from the definition found in an online dictionary^4^ was provided. In the definition, we warned the participants that Specificity does not mean Concreteness, and provided examples of a word considered to be specific but abstract (“Hinduism”), a word considered specific and concrete (“Aaspirin”), a word generic and abstract (“religion”) and a word generic and concrete (“substance”). At the end of the task, after the experimental trials, three feedback questions were asked to the participants, about the clarity of the instruction, ease of the task, and potential additional comments.

A pilot study involving ten participants was conducted before the main data collection, to test the task design and adjust the instructions. Based on the feedback received by the participants who took part in the pilot, we added the definition of specificity and the exemplar trial to the instructions.

#### Extracting specificity scores from open multilingual WordNet

Specificity ratings were extracted for 743 nouns included in both OMW and the Italian ANEW dataset (Montefinese et al., 2014).

The procedure used to extract the specificity scores is based on Bolognesi et al. (2020). Here, the authors applied three different formulas to extract specificity ratings for all the nouns included in the English WordNet 3.0 (WN 3.0, henceforth) taxonomy, relying on the semantic relation of category inclusion labeled “IS-A” in WordNet. The first two metrics, based on Resnik (1995) and an adaptation of this formula, operationalize the specificity of a word by counting the number of nodes between a word and its leaves, added to the number of nodes between that word and the top root. The third measure, which we are hereby using, calculates the specificity score of each WN 3.0 entry by dividing the amount of its direct and indirect hypernyms by the maximum depth of the WN 3.0 noun taxonomy, i.e., the maximum distance from the ENTITY root node to a leaf. Both WN 3.0 and OMW for Italian have a maximum depth of 20.^5^

### Results

We evaluated the reliability of the BWS judgements using a well-established procedure to determine consistency, namely split-half reliability (SHR). All annotations for a tuple are randomly split in two halves used to produce two independent sets of scores. Then, the correlation between the two sets of scores is computed. The higher the correlation coefficient, the more consistent are the judgements. We repeated the SHR for 100 trials, obtaining an average rho of 0.944, indicating good reliability.

Table 2 summarizes the descriptive statistics of the specificity ratings collected through BWS and the specificity scores extracted from OMW. The BWS data have been transposed into a five-point scale, for easier comparison with the OMW data, which is expressed on a five-point scale.

**Table 2:** Descriptive statistics for the specificity scores collected through BWS and extracted from OMW

Dataset	M and SD
Spec BWS	M = 3.003 SD = 0.873
Spec OMW	M = 2.478 SD = 0.603

**Fig. 1:**
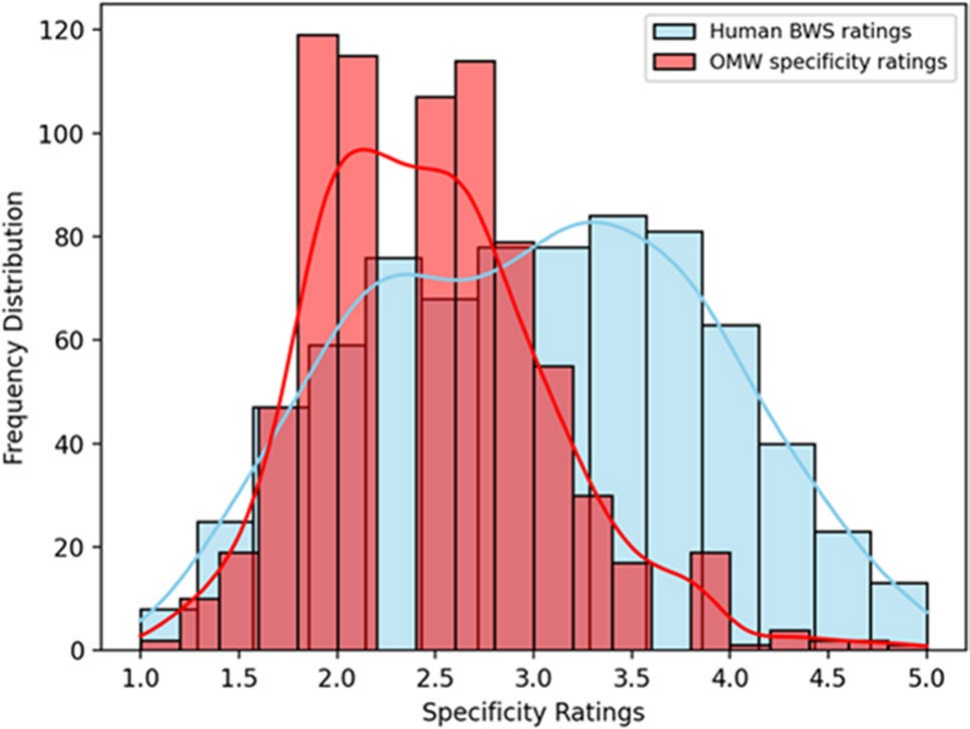
Frequency distribution of BWS ratings and OMW scores

**Fig. 2:**
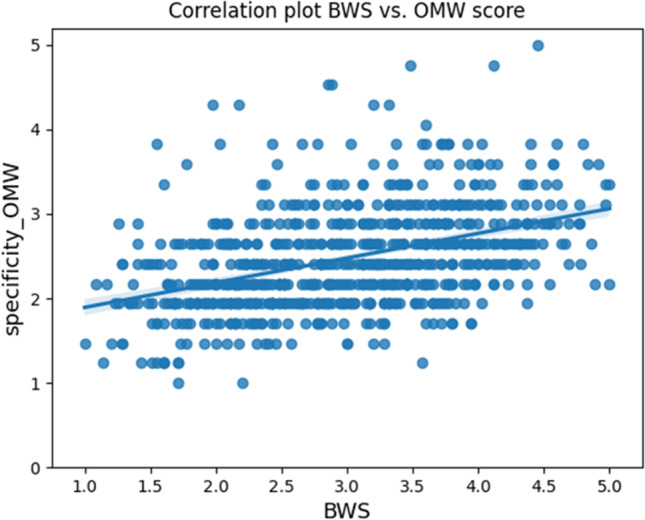
Correlation plot between BWS and OMW data

The frequency distribution of BWS ratings and OMW scores is visualized in Fig. 1. The figure shows that neither of the two datasets is normally distributed. Moreover, the two distributions are significantly different from one another as shown by a Mann–Whitney test (see analysis report in the online repository). The relation between the two variables is summarized by a significant, low positive correlation of 0.443 (Spearman correlation coefficient; *p* < 0.05), corresponding to an *R*^2^ of 0.148, visualized in Fig. 2. The interpretation of the correlation coefficients hereby reported is based on rather conservative indications exemplified by Mukaka (2012), according to which coefficients above .90 are interpreted as very high positive or negative; coefficients between .70 and .90 are high, between .50 and .70 are moderate, between .30 and .50 are low, and below .30 are very low or negligible.

The open feedback questions at the end of the survey revealed some difficulties perceived by the participants in operationalizing the task. The comments provided suggested that for some participants the task was hard because they found it difficult to understand properly what specificity meant. A few participants indicated that they felt like they had to keep in mind too many words, to make a decision about each word’s specificity and then compare with the other words. One participant, conversely, argued that the task was not too hard, because, in her opinion, words that are specific are also very infrequent. This suggests that she (and possibly other participants) may have taken a shortcut and eventually judged the words in terms of their frequency, rather than in terms of their specificity. This point will be further discussed in the General discussion section.

### Ad interim discussion

We expected to find a high, positive correlation between the Italian specificity scores extracted from OMW and the specificity ratings elicited from human participants using the BWS method. The results of this first correlation analysis show that the distributions of the two datasets are slightly different, even though they both seem to outline two peaks. These however are not as clear as in a classic bimodal distribution with two separate humps. The correlation between the two sources of specificity data is significant but not as high as we expected (Spearman coefficient: 0.443; *p* < 0.05).

This finding may be interpreted in different ways. First of all, as we already anticipated, the WN 3.0 taxonomy is informed by external knowledge bases, such as encyclopedias and other lexicographic resources. Because of the WN 3.0 many layers of in-depth knowledge in some semantic domains, such as botanics, zoology, among others, the specificity scores extracted from OMW tend to differ from the specificity scores obtained from human judgments. The in-depth lexical granularity and taxonomical knowledge featured in WN 3.0 is often not mastered by people, such as the participants in our data collection task. On average, a word in WordNet has more levels of hyponyms than the taxonomic levels present in the mental lexicon of the average speaker. Further support to this analysis can be derived by observing the plot in Fig. 2, where the majority of entries tend to be flattened on few OMW-based specificity scores while presenting a larger variation in the human judgements.

A second possible interpretation of the current findings is the following. It might be the case that specificity as a variable is particularly difficult to assess by participants, because of its relational nature. In this sense, it could be the case that participants in our study provided their judgments about the most specific and the most generic words in a tuple, using shortcuts and developing strategies that enabled them to easily fulfill the task without employing much cognitive effort (as in the case of the participant who suggested that Frequency may be used as a proxy for Specificity). We are going to delve deeper into this possible interpretation in the general discussion of our findings, in the General discussion and Conclusions section.

From this first analysis, we conclude that at least for the category of Italian nouns hereby analyzed, lexical scores extracted from WordNet to proxy specificity should be taken with caution, because such data may not always reflect with a high degree of fidelity the information encoded in our minds, and in particular the knowledge present in native speakers’ mental lexicon.

## Study 2: What is the relation between human-generated concreteness and specificity data?

### Methods

To investigate the relation between human-generated concreteness and specificity ratings we analyzed the distributions and the correlation between the dataset of concreteness ratings collected by Montefinese et al. (2014) and the dataset of specificity ratings collected through BWS method.

The dataset of concreteness ratings contains 1121 unique target words (verbs, adjectives, and nouns) in Italian, translated and integrated from the words included in the original English dataset of affective norms ANEW. *N* = 1050 words (Bradley & Lang, 1999).^6^

### Results

The distribution of specificity scores collected with the BWS method showed an average specificity of M = 3.015 (SD = 0.878). The distribution of concreteness scores collected by Montefinese and colleagues (hereby transposed from a nine-point scale to a five-point scale for easier comparison with specificity scores) have an average concreteness of M = 3.327 (SD = 1.012).

**Fig. 3:**
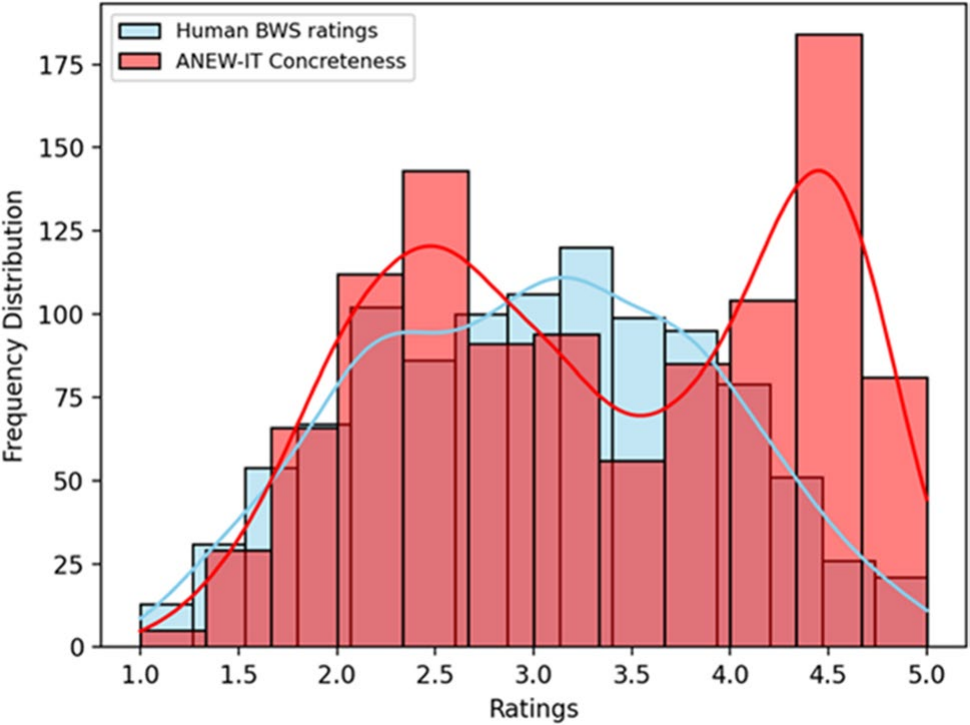
Frequency distribution of BWS ratings and Italian ANEW concreteness scores

**Fig. 4:**
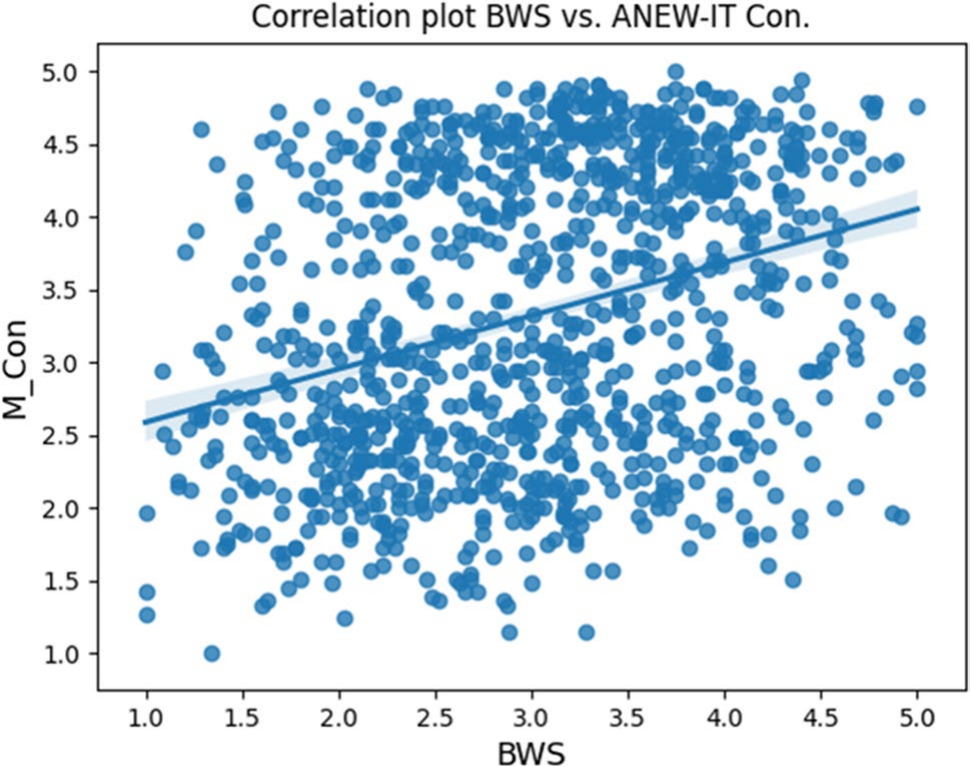
Correlation between human-generated specificity and concreteness data

The frequency distribution of human-generated concreteness and specificity ratings is visualized in Fig. 3. The figure shows that neither of the two datasets is normally distributed. Moreover, the two distributions are significantly different from one another as shown by a Mann–Whitney test (see analysis report in the online repository). The relation between the two variables is summarized by a low positive and significant correlation of 0.316 (Spearman correlation coefficient; *p* < 0.05), corresponding to an *R*^2^ of 0.1, visualized in Fig. 4. Moreover, the partial correlation between Specificity and Concreteness, when controlling for all other variables, is a very low but significant 0.163 (*p* < 0.05).

### Ad interim discussion

Overall, we expected to find a positive correlation between human-generated concreteness and specificity scores, similarly to the positive correlation found in previous analyses based on English nouns, between WordNet-derived specificity scores and concreteness ratings (see Bolognesi et al., 2020). However, we also expected this correlation to be low, as the mentioned study already showed. This would be in line with the idea that Concreteness and Specificity capture different aspects of abstraction, which are only partially correlated with one another. The first analysis confirms this hypothesis, revealing an overall low correlation of 0.316 between human-generated concreteness and human-generated specificity scores. Figure 4 supports this finding by showing that data points are spread all over the quadrants of the distribution plot.

In line with previous findings, we therefore argue that Concreteness and Specificity, even when investigated by means of human judgments, capture different phenomena involved in abstraction, and their mutual relation is not simply a linear and high correlation where words denoting abstract concepts are also more generic and words denoting concrete concepts are also more specific. The following examples may clarify the type of words that appear in each of the four quadrants, namely, (a) words that are concrete and specific; (b) words that are abstract and specific (c) words that are concrete and generic; and (d) words that are abstract and generic. The noun *muffin*, the adjective *blond* and the verb *bake* are instances of words that score high in concreteness and in specificity. Interestingly, these are words that are also fairly frequent and/or familiar and imageable. Words that can be labeled as abstract and specific, include the noun *scapegoat*, the adjective *devoted* and the verb *soothe*. Interestingly, these appear to be also quite infrequent and less familiar and imageable than the previous ones. The noun *food*, the adjective *ugly* and the verb *cook* are instances of words that score high in concreteness but low in specificity (concrete and generic). These, again, appear to be frequently used, highly familiar, possibly learned quite early, during language development, and easily imageable. Finally, words that are abstract and generic include the noun *desire*, the adjective *odd* and the verb *activate*. These seem to resemble the typical abstract concepts which, based on literature reviews, appear to be acquired later in development, are loaded with affective content and score low on imageability.

## Study 3: What is the relation between Specificity and other lexical and psychological variables associated with Concreteness or controlled while investigating Concreteness, namely Age of Acquisition, Affective content, Frequency, Familiarity and Imageability?

### Methods

We ran a series of correlation analyses aimed at understanding the relations between word specificity and the correspondent age of acquisition, affective content, frequency of use, familiarity and imageability. In line with the hypotheses described at the end of the Theoretical Background section, we investigated the validity of linear and quadratic regressions. We further ran partial correlation analyses, in which specificity and each psycholinguistic variable are correlated, while the remaining variables are used as covariate to partial out their variance.

Existing datasets of human-generated ratings for Italian words have been used for all the variables described above (Montefinese et al., 2014; Montefinese et al., 2019; Crepaldi et al., 2016). In particular:

- Age of Acquisition ratings (AoA) were extracted from Montefinese et al. (2019) (*N* = 993). The dataset comprises AoA ratings, indicating the average age at which the word is more likely to have been learned, based on participants’ judgment. The ratings consist of a mean value of the age indicated by the participants. The span is between a minimum of 1.88 (age at which the concrete noun *mom* is learned) to 14.35 (age attributed to the acquisition of the word *kerosene*).
- Affective ratings (Val, Aro, Dom) were extracted from Montefinese et al. (2014). This dataset gathers affective scores for 1121 unique Italian words. For each of these words, the dataset contains average scores for Valence (the polarity of emotional activation, ranging from very positive feelings to very negative ones and *not* the amount of affective content, where low scores would correspond to neutral words and high scores to highly emotional words), Arousal (the intensity or degree of excitement caused by an emotion), and Dominance (the degree of control an individual feels while experiencing an emotive state). We report our analysis for 1049 words as in Study 2.
- Familiarity ratings (Fam) were extracted from Montefinese et al. (2014). The author collected these ratings on a scale from 1 (very unfamiliar) to 9 (very familiar).
- Frequency ratings (Freq) were extracted from SUBTLEX-IT, a corpus containing word frequencies extracted from subtitles (Crepaldi et al., 2016). The sample size of words found in the ANEW dataset (Montefinese et al., 2014) and SUBTLEX-IT is *N* = 1049. The number of words found in the AoA dataset (Montefinese et al., 2019) and in SUBTLEX-IT is *N* = 993. The raw frequencies were transformed into a logarithmic scale.
- Imageability ratings (Imag) were extracted from Montefinese et al. (2014). The author collected these ratings on a scale from 1 (very unimageable) to 9 (very imageable).

### Results

**Fig. 5:**
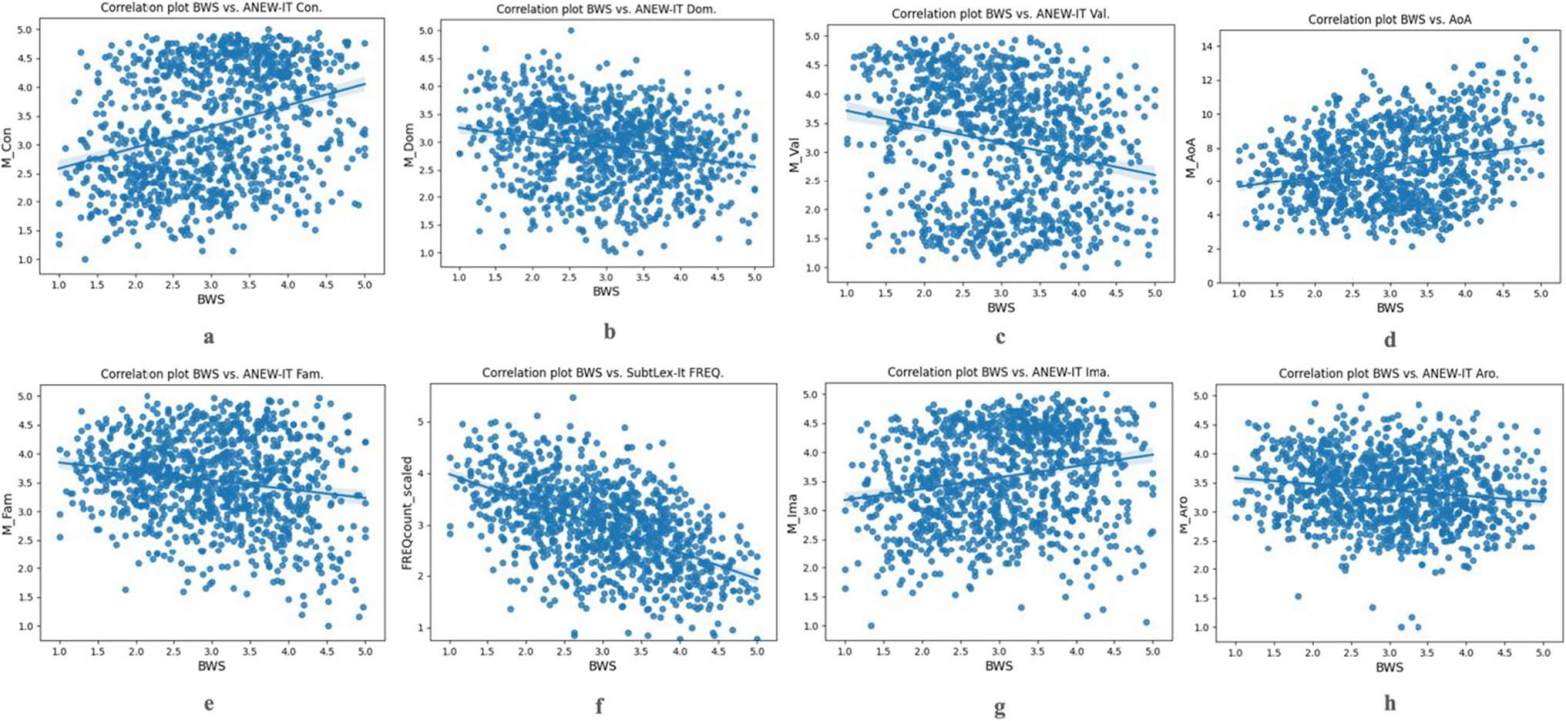
Correlation plots between specificity and related variables: **a** Concreteness (Con.); **b** Dominance (Dom.); **c** Valence (Val.); **d** Age of Acquisition (AoA); **e** Familiarity (Fam.); Frequency (SubtLex-it FREQ); **f** Imageability (Ima.); **h** Arousal (Aro.)

Table 3 reports the correlation coefficients between specificity and each of the variables taken into account, which are then displayed in Fig. 5. For AoA, Familiarity and Frequency we also tested and reported the validity of a quadratic fit, which is the type of relation that we hypothesized between each of these three variables and specificity. We observed the presence of non-linear relations on the basis of the plot of residuals for AoA and familiarity, but not for frequency. In particular, the quadratic fit for the relation between specificity data (predictor) and AoA ratings is *R*^2^ = 0.076, and for Familiarity is *R*^2^ = 0.041. The quadratic function for Specificity and AoA has a positive coefficient for the x^2^ (0.3147) showing that it is a U-shaped parabola (words with medium specificity are acquired earlier, while words with high/low specificity scores are acquired later). The quadratic function for Specificity and Familiarity has a negative coefficient for the x^2^ (– 0.1228) showing that it is an inverted U-shaped parabola (words with medium specificity have higher familiarity, while words with high/low specificity scores have lower familiarity). The linear fit between Specificity (predictor) and Frequency is *R*^2^ = 0.280, and the quadratic fit is *R*^2^ = 0.281. The Akaike information coefficient (AIC) suggests that the quadratic fit for AoA and Familiarity is better than the linear fit, whereas for Frequency it suggests that the quadratic fit is as probable as the linear model to minimize the information loss and therefore a linear model is preferred for modeling the relation between Specificity and Frequency.

**Table 3:** Correlations between specificity data and other variables, typically associated with concreteness. Significant r coefficients (*p* < 0.05) are marked with *

Psycholinguistic and lexical variables associated to concreteness		M, SD	Correlation with specificity data (BWS)	Partial correlations with specificity, and all other variables controlled
Age of Acquisition (effective age)		M = 6.964 SD = 2.255	*r* = 0.211* (linear *R*^2^ = 0.062 quadratic *R*^2^ = 0.076)	*r* = 0.157*
Affective content (scale 1–9)	Valence	M = 5.223 SD = 2.038	*r* = – 0.238*	*r* = – 0.041*
	Arousal	M = 5.630 SD = 0.912	*r* = – 0.160*	*r* = – 0.052*
	Dominance	M = 5.232 SD = 0.974	*r* = – 0.226*	*r* = – 0.038
Familiarity (scale 1–9)		M = 6.632 SD = 1.137	*r* = – 0.147* (linear *R*^2^ = 0.032 quadratic *R*^2^ = 0.041)	*r* = – 0.235*
Frequency (log)		M = 2.953 SD = 0.846	*r* = – 0.526* (linear *R*^2^ = 0.280 quadratic *R*^2^ = 0.281)	*r* = – 0.515*
Imageability (scale 1–9)		M = 6.995 SD = 1.160	*r* = 0.222*	*r* = 0.138*

The table shows a very low positive correlation between AoA and Specificity. This would suggest that the more a word is specific (e.g., *crucify* vs. *kill*) the more it tends to be learned later during development, while the more a word is generic, the earlier it is learned. However, the relationship between these two variables is best modeled by a quadratic function, suggesting that words with medium levels of specificity (basic level lexicon) tend to be learned earlier than words that are either very specific or very generic. This is in line with our initial prediction.

Regarding the affective ratings and their relation with specificity scores, valence shows a very low negative correlation score with specificity. This suggests that the more a word expresses or is associated with a positive feeling, the more the word tends to be generic (e.g., *win*), while the more it expresses or is associated with a negative feeling, the more it tends to be specific (e.g., *guillotine*). Arousal also shows a very low negative correlation with specificity. Dominance has a very low negative correlation with specificity, which disappears when controlling for all other variables (partial correlation). While it may seem that the more a word expresses or is related to feelings that individuals can control (e.g., *win*), the more the word is generic (and vice-versa for more specific words), the two variables are not related.

Familiarity shows a very low negative correlation with specificity, with a slight increase when the correlation is partial (all other variables are controlled). This would suggest that familiar words (e.g., *sleep, happy*) tend to be on average also quite generic, and vice versa, unfamiliar words (e.g., *mutilate*, *fetid*) tend to be more specific. The quadratic fit on these data, which we predicted in our initial hypothesis to be an inverted U-shape (medium specificity entails maximum familiarity) shows an advantage over the linear fit, but it explains a negligible amount of variance in the data (*R*^2^ = 0.041 as indicated above).

Frequency shows negative moderate correlations with specificity: the more a word is specific, the more it tends to be rare, while the more a word is generic, the more it is frequently used. For instance, the highly frequent noun *part*, the verb *hit*, and the adjective *good* all denote concepts that are quite generic and thus score low on specificity. Conversely, words with low frequency of occurrence, such as the noun *measles*, the verb *decompose*, and the adjective *rancid* score high on specificity. The presence of a quadratic fit on these data is not supported, suggesting that the most frequent words tend to be very generic.

Imageability shows an overall a very low positive correlation with Specificity. This suggests that words that are highly imageable (e.g., *nipple*) tend to be also highly specific.

In general, the partial correlation analyses indicate that, when all variables are controlled, the relation between Specificity and each variable weakens. This suggests that indeed Specificity captures an aspect of word meaning that is theoretically and psychometrically different from the aspect of word meaning captured by other variables. Nevertheless, since the correlation between specificity and frequency remains the highest (– 0.515) even when the other variables are controlled, we further analyze this relation in a series of regressions reported in study 3, in which we also check whether there may be collinearity issues between Specificity and Frequency.

### Ad interim discussion

We had hypothesized to find that words with medium scores of specificity would also display a lower AoA, and words with very high and very low specificity scores to have higher AoA scores (a U-shaped quadratic fit). We found that a significant 7.6% amount of variance in the AoA data is explained by Specificity: words with medium levels of specificity, which correspond to the basic level lexicon, tend to be learned earlier than words with very high or very low levels of specificity. This is in line with our prediction.

In relation to the affective ratings, we found very low but significant negative correlations between Valence and Specificity, suggesting that words loaded with negative valence are on average more specific than words loaded with positive affective content. On the other hand, words loaded with positive affective content tend to be also quite generic. This has been recently found also by Vassallo et al. (2020), and it was initially reported by Rozin et al. (2010). A possible explanation could be that positive emotions, like *happiness*, may broadly describe a situation of balance, of ‘emotional smoothness’, which has to be general and underspecified. Conversely, negative feelings and concepts loaded with negative connotations may be more specific because their granularity is precisely what breaks the balance and the smoothness of the positive polarity. ‘*The devil is in the details*’, in a way.

Arousal shows a similar trend, displaying very low but significant negative correlations with Specificity. The negative trend suggests that the more words are specific, the less their affective content is intense, but the values are so minimal that they are basically negligible. As for dominance, the presence of a relationship with specificity does not seem to be supported.

Familiarity explains 4.1% of specificity ratings in a quadratic fitted regression. The function is an inverted U-shape parabola, suggesting that words with medium levels of specificity are maximally familiar, while words with very high or very low levels of specificity are low in familiarity scores, in line with the classic literature on semantic categorization described in the Theoretical Background.

We found a moderate negative correlation between Frequency and Specificity. Our hypothesis about Frequency was to observe an inverse U-shaped distribution (best explained by a quadratic function) in which words with medium scores of specificity would correspond to the highest values of frequency, while very specific and very generic words (thus superordinate and subordinate lexicon, as opposed to the basic level) would correspond to low frequency scores. We did not observe this distribution in our data, nor the presence of a quadratic fit was supported by further analysis. The linear correlation between the two variables indicates that generic words correspond to high frequency scores and increasingly more specific words correspond to increasingly less frequent words. This does not seem to support classic cognitive semantic research that suggests that the basic level lexicon has higher frequency of use, compared to superordinate and subordinate words (e.g., Rosch et al., 1976).

Finally, in relation to Imageability, we expected to find a pattern that reflects the correlation between Specificity and Concreteness, based on the argument that Imageability and Concreteness are highly correlated with one another. We found a positive but very low correlation between Imageability and Specificity suggesting that words that are more specific are also more imageable, and probably more concrete, but there is nevertheless much room left for words that are highly specific and not easily imageable. These include illnesses such as *syphilis* and *scurvy*, or even concrete animals that we may know rather well for their behavior but we cannot picture them easily, because we rarely see them, like *termite*. These nouns appear to be also infrequent and unfamiliar. Conversely, nouns that are highly generic and quite imageable include frequent words that are highly familiar, such as *vehicle*, *people*, and *food*.

## Study 4: What is the role of Specificity in lexical access and therefore how do human-generated specificity scores predict chronometric data?

### Methods

In a series of regression studies, we analyzed: 1. the relation between Specificity and chronometric data in a lexical decision task retrieved from Vergallito et al. (2020). 2. the relation of each of the variables associated with the general phenomenon of abstraction (namely: Familiarity, Imageability, Concreteness, AoA, Valence, Arousal, Dominance, and Frequency) with chronometric data, in individual regressions. 3. the amount of variability in reaction times explained by all significant predictors together, and the fraction explained by each predictor alone, on top and beyond the variance explained by all other significant ones.

As illustrated in study 3, there is a difference in the sizes between the datasets hereby used (Montefinese et al., 2019; *N* = 993; and Montefinese et al., 2014; *N* = 1049). The reason why we used both datasets is that the former (Montefinese et al., 2019) provides AoA ratings, which are not present in the latter.

We checked whether there would be any (multi)collinearity issues among the variables used as predictors in the regressions. For all the regressions, we hereby report the *R*^2^ values (i.e., the proportion of variance of the dependent variable which is explained by the predictor(s)). The complete analyses are stored in the online repository.

### Results

Table 4 reports all the *R*^2^ for the individual regressions against reaction times. Concerning Specificity, we observe that there is an overall effect of pecificity over chronometric data (F (1, 1047) = 261.8, *p* < 0.05) that describes a considerable 20% of the variation in reaction times on the lexical decision task (*R*^2^ = 0.200). However, the plot of the residuals clearly shows a non-linear relation, as predicted by our hypothesis (Hp4). As a matter of fact, the quadratic regression, *R*^2^ = 0.227, explains a higher amount of variance.

**Table 4:** Output of the regressions over chronometric data. All variables are from Montefinese et al. (2014), *N* = 1049, except for Age of Acquisition, based on Montefinese et al. (2019), *N* = 993

Simple linear regressi﻿on - predictor	*R*^2^ of the response (RT in LDT)
Specificity	*R*^2^: 0.200 (slope β = 0.249, *p* < 0.05) (quadratic fit: *R*^2^ = 0.227, quadratic term β = 0.092)
Imageability	*R*^2^: 0.076 (slope β = – 0.116, *p* < 0.05)
Concreteness	*R*^2^: 0.024 (slope β = – 0.045, *p* < 0.05)
Familiarity	*R*^2^: 0.174 (slope β = – 0.179, *p* < 0.05)
Age of Acquisition	*R*^2^: 0.308 (slope β = 0.117, *p* < 0.05)
(log)Frequency	*R*^2^: 0.485 (slope β = – 0.174, *p* < 0.05)
Valence	*R*^2^: 0.054 (slope β = – 0.056, *p* < 0.05)
Dominance	*R*^2^: 0.034 (slope β = – 0.093, *p* < 0.05)
Arousal	*R*^2^: 0.000 (slope β = – 0.011, *p > 0.05*)

Table 4 shows also that, when considering how each individual variable relates to decision latencies in reaction times:

- Specificity has a positive effect on chronometric data (more specific words take longer to process, while more generic words are processed faster). Around 20% of the variation in chronometric data is explained by Specificity in a linear model. Moreover, the quadratic fit, as predicted, has a positive quadratic coefficient that implies that the parabola has a U-shape, and therefore words with medium specificity are processed faster than words with very high or very low specificity. In the quadratic fit, around 22.7% of the variation in chronometric data is explained by Specificity.
- Imageability has a negative effect on chronometric data (more imageable words are processed faster). Around 7.6% of the variation in chronometric data is explained by Imageability.
- Concreteness has a negative effect on chronometric data (more concrete words are processed faster). Around 2.4% of the variation in chronometric data is explained by Concreteness.
- Familiarity has a negative effect on chronometric data (more familiar words are processed faster). Around 17.4% of the variation in chronometric data is explained by Familiarity.
- AoA has a positive effect on chronometric data (words acquired earlier are processed faster). Around 30.8% of the variation in chronometric data is explained by AoA.
- Frequency has a negative effect on chronometric data (more frequent words are processed faster). Around 48.5% of the variation in chronometric data is explained by Frequency.
- Valence has a negative effect on chronometric data (positive words are processed faster than negative words). Around 5.4% of the variation in chronometric data is explained by Valence.
- Dominance has a negative effect on chronometric data (more dominant words are processed faster). Around 3.4% of the variation in chronometric data is explained by Dominance.
- Arousal is not a significant predictor of chronometric data, in line with previous studies (e.g., Larsen et al., 2008).

Finally, we fitted a model where all variables but Imageability are used as predictors and decision latencies (lexical decision’s reaction times) are used as the dependent variable. We excluded Imageability because it shows collinearity with Concreteness (VIF = 6.377 using data in Montefinese et al., (2014); VIF = 6.769 using data in Montefinese et al., 2019). In Table 5, we report the results of these models, based on Montefinese et al. (2014) (*N* = 1049), and in Table 6 the results based on Montefinese et al., 2019; *N* = 993, which includes AoA as a predictor. Once we had the total amount of variance in decision latencies (expressed by the *R*^2^) explained by all (significant) predictors, we dropped each predictor in turn from the model (i.e., ablation) and calculated the difference between the *R*^2^ values, which describes the fraction of variance in reaction times explained uniquely by the dropped predictor.

**Table 5:** Regression for the lexical decision task for the Montefinese et al. (2014) data with all variables and ablation. Ablation has been conducted only for the significant covariates (*p* < 0.05) - marked with an *asterisk* in the table

Model	β value	*R*^2^	Delta Δ
All Variables:		0.532	-
• Frequency • Familiarity • Concreteness • Specificity • Valence • Arousal • Dominance	– 0.1394* – 0.0704* – 0.0198* 0.0811* – 0.0036 0.0018 0.0096		
- Frequency	–	0.334	0.198 (19.8%)
- Familiarity	–	0.516	0.016 (1.6%)
- Concreteness	–	0.529	0.003 (0.3%)
- Specificity	–	0.521	0.011 (1.1%)

**Table 6:** Regression for the lexical decision task for the AoA data (Montefinese et al., 2019) with all variables and ablation. Ablation has been conducted only for the significant covariates - marked with an *asterisk* in the table

Model	β value	*R*^2^	Delta Δ
All Variables:		0.565	-
• Frequency • Familiarity • Concreteness • Specificity • Valence • Arousal • Dominance • AoA	– 0.1217* – 0.0522* – 0.0009 0.0559* 0.0017 – 0.0137 – 0.0007 0.0463*		
- Frequency	–	0.423	0.142 (14.2%)
- Familiarity	–	0.557	0.008 (0.8%)
- Specificity	–	0.557	0.008 (0.8%)
- AoA	–	0.541	0.024 (2.4%)

The results in Table 5 show that the overall *R*^2^ explained by Frequency, Familiarity, Concreteness and Specificity is 0.532 (53.2% of the variability in decision latencies). Valence, Dominance, and Arousal are nonsignificant predictors. All significant predictors besides Specificity have negative beta values. This suggests that Frequency, Familiarity, and Concreteness have a negative effect on chronometric data (the higher each of them is, the faster the words are processed) whereas Specificity has a positive effect (the higher the specificity, the slower the words are processed).

In Table 6, where we replicated our model on a different dataset (Montefinese et al., 2019) to include AoA as a predictor, we observe a similar behavior, with minor differences. In this case, we have a global *R*^2^ of 0.565 (56.5% of variability in decision latencies explained). Like in Table 5, we observe that Valence, Dominance and Arousal are nonsignificant. In addition to them, Concreteness also qualifies as nonsignificant in this model. The behavior of the significant predictors is the same: Frequency and Familiarity have negative Beta values, while Specificity has a positive value. AoA also qualifies as a positive predictor, indicating the AoA has a positive effect (the higher the age of acquisition, the slower the words are processed).

### Ad interim discussion

Frequency explains much of the variance in chronometric data: frequent words are recognized faster than infrequent words. This is not big news. Moreover, AoA explains a considerable amount of variance in lexical latencies, with early acquired words being responded to faster than later-acquired words. This, as well, has been previously reported (e.g., Cortese and Khanna, 2007). What, instead, we add as an interesting finding is that in the first of our models Specificity explains a good 20% of the variance in lexical decision latencies. When a quadratic fit is tested, then Specificity explains almost 23% of the variance in chronometric data in a simple regression. This suggests that words with medium specificity scores, which Rosch would label as basic level lexicon, indeed tend to be recognized faster than words that are associated with very high or very low specificity scores. Furthermore, our analyses indicate that Specificity is solid from a theoretical as well as from a psychometric point of view.

Finally, when all variables are included in a model as predictors for chronometric data, it appears that all dimensions of affective content (Valence, Arousal, and Dominance) as well as Concreteness (when AoA is included) are non-significant predictors. The remaining variables, namely AoA, Frequency, Familiarity and Specificity together explain 56.5% of the variability in decision latencies. While Frequency and AoA remain the strongest predictors, Specificity and Familiarity as well explain a unique fraction of these behavioral data. In particular, Specificity accounts between 1.1% and 0.8% of the variability above and beyond all the other predictors, in decision latencies. This makes Specificity a strong predictor.

## General discussion and conclusions

The analyses hereby presented and discussed are based on a sample of around 1K Italian words, for which we collected specificity scores using Best-Worst Scaling. This method has been recently shown to produce semantic norms with higher predictive validity than other response formats such as rating scales and numeric estimation (Westbury & Hollis, 2018). As Westbury and Hollis show, the BWS method produces norming data that are also qualitatively different from data produced with other methods. The reason for such differences remains to be understood, and can probably be found in the type of semantic information about word meaning that participants activate when they are asked to perform the specific task required by the experimental design. In general, however, the BWS implies that words (and concepts) are *compared* to one another, in their relation to a variable of interest. We found this peculiarity well suited to the nature of our investigation and for the variable that we analyze. The purpose of our data collection was to collect specificity data, namely, to understand how people rate the inclusiveness of a category denoted by a linguistic label. This could be intended as a human-generated judgment about categorical size, which can be metaphorically expressed as ‘how big is the container (namely, the category) denoted by a linguistic label’. For instance, the ‘container’ for the category *food* is quite big, because such a category can accommodate several different members, while the ‘container’ for *pomegranate* is smaller. An educated estimate about category size, or any size, is intrinsically a relative metric. The size of a conceptual category is relative to the size of other categories. The category *pomegranate* is smaller, or better, less inclusive, and thus more precise than the category *food*. Similarly, the category *food* is smaller than the category *objects*. Specificity in this sense is a relational property. For this reason, we argued, it is better tackled with a method that enables participants to *compare* different words with one another. The BWS method fulfills this purpose because it requires participants to indicate the best and the worst item within a group of four, in relation to a variable of interest, namely Specificity. We believe that other variables, such as for instance conceptual Concreteness, may be tackled appropriately by other methods. Concreteness, in fact, is a property of the referent denoted by a conceptual category (and its related word). This is rather an absolute property of a word/concept, which can be evaluated without resorting to the concreteness of other words. Concreteness is much less inherently relativistic, compared to Specificity.

Having adopted BWS for the purpose of our data collection, and having defended its theoretical soundness for the variable hereby investigated (namely, Specificity), does not make us blind toward the potential limitations of this method, for our data collection. As described in the Results section of Study 1, some participants found the task quite difficult, suggesting that Specificity is indeed a complex variable to judge, despite the fact that in the instruction we provided a dictionary-based definition and an example of how the task would look like. The main reason for such perceived complexity could be found in the cognitive load that participants undertake while judging a word in relation to its specificity, in a context where words do not belong to the same lexical field. As a matter of fact, determining whether a word is more or less specific than another word, even when using the BWS method, may push participants to mentally form a sort of taxonomy in which several categories need to be mentally identified and organized before expressing a judgment. Critically, one participant mentioned the perceived correlation (which indeed was supported by our analyses, although it is not problematic in terms of collinearity) between Frequency and Specificity, suggesting that she might have developed a strategy to easily fill the task by indicating within each tuple the most frequent and the least frequent word, instead of the most generic and the most specific ones. In this sense, a follow-up study on this topic could use different methods to investigate and replicate the high negative correlation found between Specificity and Frequency.

The low positive correlation observed between the Specificity collected with BWS and the specificity scores extracted from WordNet suggests that the hierarchical relations of category inclusion encoded in WordNet do not reflect accurately those in the mind of human speakers, or at least of the participants who took part in our data collection, namely, a large group of mostly undergraduate Italian students. As previously mentioned, some semantic domains in WordNet are excruciatingly detailed, because external knowledge bases that include specialistic terminology have been used to implement the WordNet taxonomy. In this sense, WordNet behaves as a language expert in all semantic domains, while different groups of speakers may be experts in some fields but not in others. For instance, experts in the field of biology, veterinary and natural sciences may master a much wider range of terms related to the animal kingdom, compared to experts in chemistry or modern languages. As a consequence, when the name of an animal species (e.g., *horse*) is mentioned in the former group, it may be perceived as a rather generic word, if the speaker is an expert in horse breeds. Conversely, this category may be perceived to be already quite specific to the non-expert. Expertise, therefore, may very likely affect word specificity. In this regard, further studies may collect specificity data from speakers, together with demographic information about the participants. In this way, it would be possible to observe how word specificity may change, depending on whether the participants to the data collection are adults vs. children, experts vs. non-experts, avid readers vs. lazy readers. The authors are currently tackling this issue, within the project ABSTRACTION (ERC-2021-STG-101039777) (https://www.abstractionproject.eu/). 

The relation that we observed between Specificity and Concreteness, the core question of our investigation, confirms our hypothesis, showing a correlation coefficient that is positive but low. As previously shown with WordNet data (Bolognesi et al., 2020), Concreteness and Specificity capture two different aspects involved in abstraction. The two aspects are correlated at a low level, with abstract words tending to be also more generic and concrete words more specific. The relation is rather complicated and actually there are also words that denote abstract and specific categories and words that denote concrete but generic categories. It is therefore important to keep the two variables disentangled. As mentioned in the Introduction, previous studies on word concreteness typically control for differences in some psycholinguistic variables, such as word frequency between concrete and abstract words, but not for other variables. Crucially, none of the studies focused on the differences between concrete and abstract words and concepts controls for word specificity because of the lack of lexical resources that could be used to operationalize this variable. Our study addresses this gap and provides a lexical resource of specificity scores generated by humans, which can be used in future psycholinguistic investigations.

The relation between Specificity and other psycholinguistic variables shows interesting points: first of all, frequency appears to be negatively correlated with specificity (more frequent words are more generic, less frequent words are more specific). The two variables are not collinear. The correlations between specificity and other psycholinguistic variables excluding dominance are very low (positive or negative).

Most interestingly, we showed that Specificity in individual regression over chronometric data explains 23% of the variance in decision latencies during lexical decisions. This result, derived from a quadratic regression, suggests that words with medium levels of specificity are the easiest to process. The relation increases to explain around 56.5% of decision latencies when considering AoA, Frequency, Familiarity and Specificity together. Moreover, Specificity alone, on top and beyond all the other significant predictors, could account for around 1% additional variance, which makes it a strong predictor, from a psychometric point of view.

These findings support our claim about the importance of word specificity in language processing and word meaning representation and our advice to control for specificity in psycholinguistic experiments. As indicated in the Introduction and Theoretical background of this work, studies focusing on concreteness have shown some contrasting findings which, we argue, may be attributed to the fact that the stimuli were not controlled for specificity. Further studies can therefore attempt to conceptually replicate the concreteness effect reported in the literature, using samples of concrete and abstract words that are balanced in specificity.

Overall, the implications of these findings for theories and models of word processing and representation are multiple. First, the relation between Specificity and decision latencies sheds some light on the semantic richness effect reported by previous empirical studies (e.g., Pexman et al., 2002; Pexman et al., 2003; Grondin et al., 2009). These studies are based on concrete nouns only, typically belonging to the basic level vocabulary and show that words characterized by a high number of features are processed faster than words characterized by few features. With our results we show that words with medium levels of Specificity (that is basic level vocabulary) are easier to process than highly specific and highly generic words. Our sample includes both, concrete and abstract words.

 To conclude, our findings support the importance of semantic features in conceptual representation and suggest that words varying in specificity may vary also in their featural configuration. This opens up new questions, in relation with the type of features that characterize the representation of specific vs generic categories and, consequently, the type of conceptual representation (e.g., prototype-based or exemplar-based) afforded by categories that vary in Specificity. We hope, with the present set of studies and related dataset of specificity data, to raise a new wave of empirical studies aimed at collecting specificity data for larger samples of words in multiple languages and therefore advance and improve the investigation of the general phenomenon of conceptual abstraction.

## Footnotes

1 This refers to the entry *insect* defined as "small air-breathing arthropod".

2 BWS has been used to collect human judgements for age of acquisition, valence, arousal, and concreteness (Hollis & Westbury, 2018), sentiment scores of lexical items (Kiritchenko and Mohammad (2017), among others.

3 http://www.saifmohammad.com/WebPages/BestWorst.html

4 https://www.treccani.it/vocabolario/specificita

5 Access to WN 3.0 and the Italian portion of OMW has been obtained using the Python library NLTK v. 3.5.

6 An additional amount of five words could not be automatically retrieved.
